# Assessment and feedback in dental education: a journey

**DOI:** 10.1038/s41415-022-4968-1

**Published:** 2022-09-23

**Authors:** Vince Bissell, Luke J. Dawson

**Affiliations:** School of Dentistry, Institute of Life Course and Medical Sciences, University of Liverpool, Liverpool, UK

## Abstract

The authors describe their personal experience of responding to changing perceptions of best practice and the expanding evidence base, in relation to assessment and feedback in dental education. Changes at a particular dental school over the years are described, along with a more general outlook, culminating in suggestions for future directions.

## Key points

- Assessment in dental education has changed considerably in the past 20-30 years, at least partially driven by developments in assessment theory and consequent concerns about the validity of traditional approaches.
- Objective assessment methods have gained considerable popularity, with apparently good reason. However, these approaches do not always allow authentic assessment of real-world clinical practice.
- Workplace-based assessments have been adapted for use in undergraduate settings. Such methods require the acceptance and management of expert judgement. They also require sophisticated approaches to the interpretation of large datasets. Properly implemented, they can facilitate defensible progress decisions but their role in supporting development is equally important.

## Introduction

In the early years of this century, significant changes in assessment were enacted in a number of dental education settings, including dental schools, in the United Kingdom. This process was, to a large extent, influenced by similar changes in medical education, although dentistry was somewhat behind the curve. It was driven by theoretical concerns about the suitability of assessment approaches that had been commonplace up until that time. The School of Dentistry in Liverpool was no exception in this regard and introduced major changes in examinations. However, examinations, useful as they may be, are of limited value in measuring clinical performance and for the past decade, the school has developed and sought to refine sophisticated approaches to longitudinal assessment that could address such issues. In this piece, we briefly describe this journey of development and seek to explain its basis in terms of knowledge, current at the time.

## Ancient history

Prior to the changes alluded to above, assessment in many dental schools, including Liverpool, typically consisted of written examinations, often essay-based and clinical examinations, that may have included unseen cases, case presentations, practical or procedural examinations, vivas and so on. Attainment of clinical competence, if it was considered in those terms at all, was a matter for those examinations to determine and to be inferred also from numbers of procedures undertaken by students during the course of their clinical studies. Such assessment approaches have very well-recognised problems, among which: they may fail to adequately sample the curriculum; they can be highly subjective and involve uncontrolled bias in marking; and the clinical components are difficult to standardise, meaning different candidates get exams of variable difficulty. Taken together, these issues (and some not discussed) raised serious problems with respect to assessment utility.

Assessment utility was introduced as a concept by Van der Vleuten in 1996,^1^ who proposed that the usefulness of an assessment method was a product of its reliability, validity, educational impact, acceptability and cost. It was recognised that no single assessment method would ever have perfect utility and that there would be trade-offs between these variables, with compromises made dependent upon the tool selected and the context in which the assessment was expected to function. Nonetheless, when the model is applied to the traditional assessment methods described above, major concerns become evident.

No discussion of assessment in medicine or dentistry would be complete without mention of Miller's famous pyramid.^2^ The framework proposed by Miller became extremely influential and helped crystallise the idea that different assessment methodologies were suited to measuring different facets of competence. In particular, it was pointed out that those methods appropriate for testing knowledge and its theoretical application (written tests) were inadequate for assessing candidate performance in a clinical context.

## The era of objectivity

And so, at the School of Dentistry in Liverpool, as elsewhere, written examinations became almost entirely of the objective variety, that is, multiple choice questions of the 'single best answer' or 'extended matching item' type. Such questions are 'objective' in that they have only one correct (or best) answer; they are easily marked but difficult to write (at least, good questions are). A typical examination paper might consist of 1-300 questions, enabling effective sampling across the full breadth of the curriculum. This latter point was seen to be important in relation to both reliability and validity and we adopted the very useful approach of 'blueprinting' to ensure that all the learning outcomes had been assessed at some point in the assessment scheme, using an appropriate method (as illustrated by Miller's pyramid).

The changes introduced also incorporated understanding, current at the time, of the importance of setting defensible passing standards using acceptable and diligently applied methods, such as Angoff, Hofstee, Borderline Group (for performance assessments) and variants thereof.^3^

Objectivity in relation to assessment of demonstration of clinical performance (the 'shows how' of Miller's pyramid) was pursued by the introduction of objective structured clinical examinations (OSCES), much 'in vogue' in medicine at the time. The approach taken to OSCEs was common then and remains so. Stations were designed to test a range of clinical skills, with the actual patients featured in traditional unseen cases replaced by standardised role-players. The tasks set were broken down into their component parts, facilitating marking with a checklist as each part was seen to be 'done', 'partially done' or 'not done' - this was the objective element of the process.

It is important to point out that there are other approaches to OSCE delivery, for instance, the use of global rating scales aligned to clinical skills domains rather than checklists. Such an approach allows aggregation according to domain, which seems inherently more sensible than aggregation within assessment instruments. However, a particular limitation of OSCEs, whatever the marking and aggregation scheme, is that they are not designed to assess what happens in the real world of clinical practice; the 'does' of Miller's pyramid. This is the sphere of the workplace-based assessment (WBA). We have previously set out the need for highly sophisticated approaches for the determination of competence^4^ and provided evidence that suggests that reliance of numbers of procedures completed is not valid.^5^ We would suggest that one-off tests of competence are also fraught with difficulty when considering their validity. The school recognised these issues from the beginning of the changes described and sought to develop a system of continuous longitudinal assessment and feedback, anchored on a judgement related to the degree of independence with which a procedure was undertaken, that would allow the concepts of WBAs to be deployed in the undergraduate setting. A key feature of the system was that it was mapped to multiple sets of learning outcomes, including, at the time, those of *The First Five Years* and the vocational training curriculum. Collection of assessment data quickly moved from paper to a digital system linked to a database, allowing the thousands of data points collected in relation to each student to be viewed, analysed and interpreted. In theory, therefore, it was possible to determine a student's development trajectory over time and the point at which they had reached an acceptably consistent level of independent performance in relation to particular clinical skills.

## 'Programmatic' approaches

We would contend that the system of continuous longitudinal assessment used in our school is an attempt to operationalise programmatic assessment (PA), broadly aligned with principles suggested by the proponents of that assessment philosophy,^6^ aspects of which are worthy of brief consideration here.

Students are assessed in relation to each patient and simulation encounter. There is no doubt that this can be perceived as stressful by students and consequently, by the staff responsible for undertaking it. It is vital, therefore, that students and clinical supervisory staff recognise that these daily episodes of assessment are very much 'low stakes' to support 'learning moments';^6^ taken alone they have little significance for the student's progress. Of much greater importance than the assessment of performance taking place is the developmental feedback provided. In other words, this is assessment for learning.

Of course, progression and certification decisions must be made at some point. In PA 'high stakes' decisions require a level of data proportional to the stakes of the decision.^7^ Therefore, very many data points need to be brought together for each student. This represents one of the most significant difficulties in putting the theory of PA into practice. Longitudinal assessment systems of the type described can generate many thousands of data points for each student and each of these points may have rich, associated, contextual data. Discerning patterns and making holistic judgements using such data is undoubtedly challenging. We fully accept the view that expert judgement, with its inherent subjectivity, is essential throughout the assessment process and in the task of interpreting very many items of low stakes data.^6^ With this in mind, we employ a staged process set out in a standard operating procedure, with clinical academics reviewing longitudinal, discipline-specific data before a meeting of a multidisciplinary panel of experts. Our experience has been that, for the vast majority of students, the data usually indicates satisfactory progress, but, for a small number, the decision will be less straightforward and it is for these students that all available information must be brought to bear in occasionally lengthy deliberation. Van der Vleuten *et al*.^6^ have described an 'intermediate evaluation' *en route* to the final decision, providing for a 'remediation moment'. We employ just such a process, with one or more Clinical Development Monitoring Panels taking place in each year of studies, providing detailed feedback to students, and the final Clinical Assessment Panel tasked with making decisions on progress and final graduation.

## A brief word on feedback

We do not propose to say a great deal about feedback, other than to note that despite the fact that students profess that they want it, in large volumes, they often don't seem to 'hear' it or be capable of engaging with it to great effect. This is not a new observation and there is an extensive literature on the subject.^8^ We have been influenced by evidence suggesting that long-term mentoring relationships could be important in facilitating responsiveness to feedback.^9^ Consequently, our academic advising system, in which students are allocated to an advisor for the duration of their studies, involves reflective writing and discussion based on feedback from multiple sources, including the accumulating data from WBA.

## The future

**Fig. 1:**
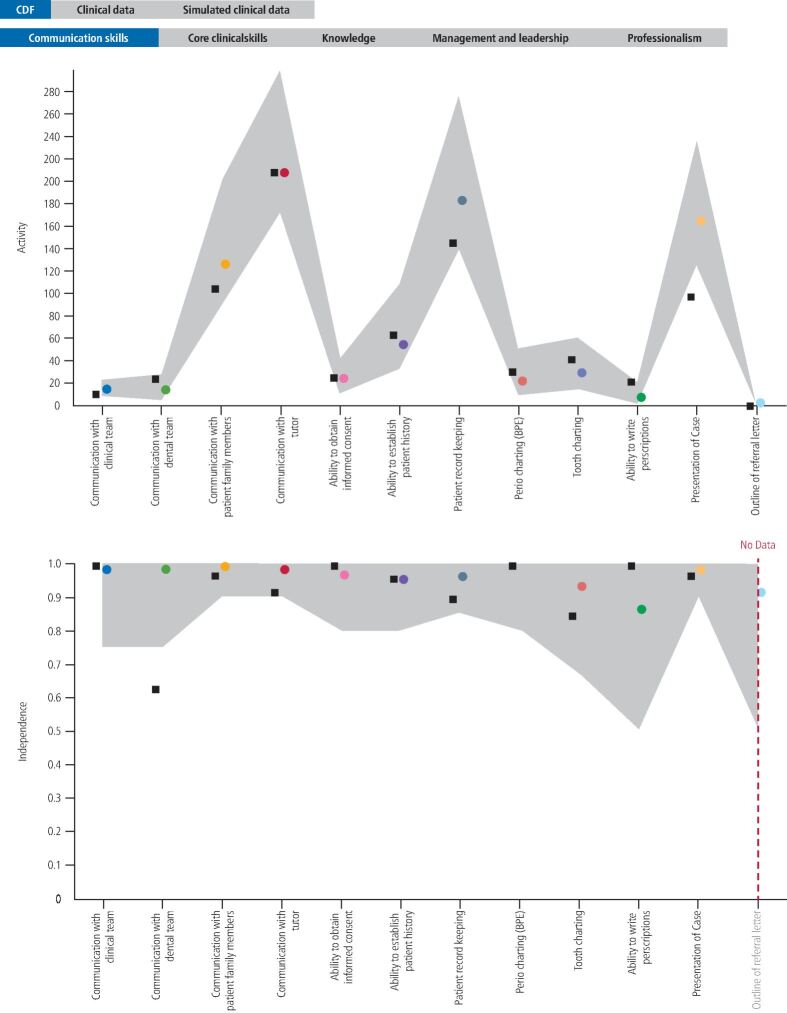
Shows activity and independence ribbon plots for the communication skills domain. The grey ribbons represent the reference range calculated from graduating students from three complete cohorts, the coloured dots show the mean of the range for each individual competency and the black squares show the current development of a specific student. The activity data are cumulative. However, the independence data are year-specific as our research^5^ shows that independence changes significantly from year to year. This means that the independence plot also shows if there has been activity in the current year of study. Black squares outside of the reference range suggest the need for more detailed examination of associated contextual data

As alluded to above, expert judgement is essential to all stages of the assessment process and any attempt to remove it in an effort to achieve complete objectivity is only likely to lead to trivialisation of the assessment process.^6^ However, reliable data will always be essential to good assessment and we would contend that there is a role for technology, not just in capturing the data, but in facilitating its organisation and display for meaningful interpretation. Within the school, a team of academics and programme developers have been working on an approach to visualisation that will allow the data of individual students across multiple domains to be viewed against a reference range (Fig. 1). This would allow areas of performance lying outwith historical norms to be quickly identified, not to serve as conclusive evidence for a final decision, but to flag the need for a more granular examination of context-rich data, as well as serve as developmental feedback for students to assist with goal setting and deliberate practice.^10^

We are very much aware that there are important developments in assessment with which we have only tangentially engaged at this point, one such being the concept of entrustability.^11^ The articulation of entrustability decisions, which are based on holistic clinical encounters, with our programmatic approach, is something we intend to investigate as a further step in relating assessment outcomes to real world capability. With reference to Figure 1, entrustability approaches require moving from triangulating and integrating data for single skills in a single domain to triangulating and integrating data for the entire clinical encounter in the context of which the skill is being undertaken.

These conceptual choices bring us to the question: how will we know what new approaches are useful? In recent years, we have been persuaded that the answer to this question can best be seen through the lens of argument-based validity, particularly the model proposed by Kane.^12^ We suggest that progress in assessment will be dependent upon rigorous validation through this type of approach. The implications of our assessment decisions are of such importance that the methodology must not escape serious scrutiny. Furthermore, it is essential to also consider educational impact. Focusing on assessment is known to be problematic, leading to many unintended and unfortunate consequences in student behaviours.^13^ However, presenting sophisticated assessment approaches to students within a framework of development and then requiring them to focus on acting to address their identified development needs would be a more authentic driver of progression. Moreover, progression on this basis is much more in keeping with an ethos of lifelong learning, compared with evidence based on reaching a pass score on a standardised test. After all, if students are simply working to pass tests, what happens to their development when the tests are gone?

## Conclusions

Anyone involved in the transition from undergraduate dental education to foundation and vocational training will recognise this as a sphere of contention. There is much debate about the nature of the 'safe beginner' and the most appropriate means of demonstrating that this status has been achieved, with, not surprisingly, the General Dental Council taking a particular interest. The debate has only intensified over the past two years as the COVID-19 pandemic has taken its toll on pre- and post-graduation training. The importance of evidence-based assessment in this context cannot be over-stated. However, there are two final points to make, of some importance we feel. The first is that no assessment process, however well-thought-out and validated, will be infallible. Wherever the standard is set, there will always be borderline cases and judgements will need to be made. The second is that assessment is not the whole story, or, for that matter, the story's end. We should see assessment as providing information about learners that: a) points to their progress along a continuum of professional development; and b) suggests what they need to do to move forward on that journey. Putting this philosophy into action seems essential if we are to engineer transitions in our training pathways that serve both professionals and patients.
